# Machine Learning Applications for Venous Ulcer Assessment and Wound Care: A Review

**DOI:** 10.3390/diagnostics16030373

**Published:** 2026-01-23

**Authors:** Miloš Madić, Nikola Vitković, Zoran Damnjanović, Sanja Stojanović

**Affiliations:** 1 Department of Production and Information Technologies, Faculty of Mechanical Engineering, University of Niš, 18000 Niš, Serbia; nikola.vitkovic@masfak.ni.ac.rs; 2 Department of Surgery and Anesthesiology with Reanimatology, University of Niš, 18000 Niš, Serbia; zoran.damnjanovic@medfak.ni.ac.rs; 3 Clinic for Vascular Surgery, University Clinical Center Niš, 18000 Niš, Serbia; 4 Department of Biology with Human Genetics, Faculty of Medicine, University of Niš, 18000 Niš, Serbia; sanja.stojanovic@medfak.ni.ac.rs

**Keywords:** venous ulcers, chronic wounds, machine learning, wound care

## Abstract

Over recent years, venous ulcer wound care has experienced significant advancements through the application of machine learning (ML) models. The aim of the present study is a systematic, comprehensive analysis of prior research studies in this field covering the period between 2001 and August 2025. By searching multiple academic databases, including the Web of Science, Scopus, and PubMed, using relevant keywords and different queries, and screening reference lists of previously published manuscripts and review papers with a focus on the application of artificial intelligence in dermatology and medicine, an initial set of potential studies for review was obtained. To ensure the scope and relevance of the review, several inclusion and exclusion criteria were used to derive the final set of relevant research studies upon which a database for research data management was created. As a result, a total of 79 relevant research studies were comprehensively analysed, upon which detailed meta-analysis and analysis of application areas of ML models within venous ulcer wound care were conducted. Afterwards, a summary of benefits for medical systems and patients was given along with a general discussion regarding ML model limitations, trends, and opportunities, as well as research studies’ limitations and possible future research directions. The presented analyses may be valuable for researchers interested in applying ML models not only to venous ulcer wound care but also to other types of chronic wound care.

## 1. Introduction

A wound represents a fundamental disruption in the body’s protective barrier, which is a condition wherein a normal skin structure is violated to any degree [1], emerging from various pathological operations that can originate either externally or internally in any human organ [2]. The classification of wounds can be made based on different criteria [3]. However, the most clinically significant classification system divides wounds into two primary categories based on healing characteristics: acute and chronic wounds. While acute wounds are naturally healed in a short period of time, chronic wounds are characterized by their failure to proceed through the normal healing period, typically from 4 to 12 weeks [4,5,6]. This deviation from the normal healing process typically occurs due to an underlying pathology that prevents or delays the healing process [7].

The major types of chronic wounds include venous ulcers, arterial ulcers, diabetic foot ulcers, and pressure ulcers, with less common types including surgical, malignant, and inflammatory wounds. Venous ulcers are among the most common chronic wounds [8,9], with chronic leg ulcers being the most prevalent form, comprising approximately 70% to 90% of cases [10]. The pathophysiology of venous ulcers primarily involves chronic venous insufficiency caused by the improper functioning of venous valves [8]. Additional contributing factors include neuropathy, lymphedema, trauma, rheumatoid arthritis, vasculitis, sickle cell anemia, osteomyelitis, cutaneous tumors, and infectious diseases [10]. While valve dysfunction is the central underlying mechanism, multiple demographic, occupational, medical, and lifestyle-related risk factors contribute to the development of venous ulcers. These include deep vein thrombosis, obesity, smoking, physical inactivity [11], overweight, pregnancy, prolonged standing, unhealthy lifestyle habits, unbalanced diet, hormonal changes, age over 40, genetic predisposition, leg injuries [12], and occupations with long-standing hours such as teaching, manual labour, and nursing [13].

Venous ulcers represent a significant subset of chronic wounds with well-documented prevalence patterns globally. They have a prevalence of about 1% of the adult population all over the world, encompassing rural and urban regions across both developed and developing countries [14]. In addition, venous ulcers demonstrate particularly concerning recurrence patterns that significantly impact long-term healthcare utilization. According to Chan et al. [15], venous ulcers have a 1-year recurrence rate of 25% to 50%, posing a substantial socioeconomic burden. Results from British studies indicate that between 90,000 and 108,000 patients have leg ulcerations at any one time and that three to four times this number are at risk of recurrence [16]. This finding indicates that for every active ulcer case, three to four additional individuals remain at high risk for ulcer recurrence, creating a substantial at-risk population requiring ongoing surveillance and preventive care of venous ulcers.

Chronic wounds, and venous ulcers in particular, exert profound and multidimensional effects on patients that extend well beyond the local tissue damage. The impact encompasses physical, psychological, social, and economic domains, creating a complex web of interconnected consequences that significantly diminish the overall quality of life. This multifaceted burden creates cascading effects that influence virtually every aspect of patient existence. Venous ulcers are at increased risk of complications and infections [17], including infections of the bone (osteomyelitis) or infections that spread through the bloodstream (bacteremia) and, in severe cases, gangrene and amputation [18]. In addition, patients with venous ulcers are at risk of reduced life expectancy [19].

Likewise, venous ulcers place a significant burden on healthcare systems globally, presenting complex, multi-dimensional challenges that go well beyond the direct costs of treatment. The burden encompasses financial constraints, resource allocation challenges, workforce demands, and system capacity limitations that affect healthcare delivery across multiple levels of care. A study from 1999 showed that home healthcare, hospitalizations, and home dressing changes accounted for an average total medical cost of approximately USD 10,000 per patient [20]. The assessment and management of venous ulcers require specialized expertise, diagnostic capabilities, and treatment modalities that create specific resource demands for healthcare systems. These requirements include compression therapy services, vascular diagnostic capabilities, and specialized wound care expertise that may not be available in all healthcare settings.

Due to its implications for patients, as well as the available resources and capabilities of medical centers, venous ulcer wound care is recognized as an important area of healthcare. The management framework for venous ulcers exemplifies the complexity of chronic wound care. As noted by Chan et al. [15], the management of venous ulcers requires multidisciplinary teams of doctors, nurses, and allied health professionals to ensure successful wound care, including assessment, treatment, monitoring, and ongoing management. For venous ulcers specifically, treatment options include surgery (coil embolization, endovenous laser ablation, etc.) and non-surgical treatments (compression therapy, antibiotics, pharmacotherapy, exercise, etc.) [12]. Appropriate wound care management, through the selection of the most effective therapeutic approach, can shorten healing time and reduce the treatment costs, benefiting patients physically and potentially easing the economic burden on the healthcare system [21]. In contrast, inadequate chronic wound care may hinder the identification of optimal treatment strategies and the wound healing process [22], and consequently increase both patient suffering and healthcare expenditures.

The complexity of venous ulcer wound care highlights the potential role of artificial intelligence (AI) and machine learning (ML) to support clinical decision-making, improve wound assessment, and optimize treatment strategies. ML models are capable of analysing large and complex datasets to support early prediction of wound healing, automate wound image analysis, and facilitate personalized treatment planning. In that way, it is possible to enhance the accuracy of wound assessment, reduce inter-observer variability, and enable timely interventions, especially for non-healing or slow-healing wounds. Furthermore, by promoting diagnostic consistency and efficient resource utilization, the application of ML models in venous ulcer wound care has the potential to reduce treatment costs and alleviate the burden on healthcare systems.

Great possibilities and advantages, as well as the remarkable potential and popularity of ML models, have made an increasing number of researchers use AI and ML models in venous ulcer wound care research. Therefore, this paper aims to provide a comprehensive review of the application of ML models in venous ulcer wound care by systematically analysing research and development efforts in this field. This study is organized into six sections. Following this introduction, Section 2 provides details of the conducted review methodology. Section 3 presents a meta-analysis of research studies based on year of publication, country of origin, and data type. Section 4 identifies the six key application areas of ML models in venous ulcer wound care and provides for each a summary with essential details related to the development of ML models and data characteristics. This section also discusses the benefits for patients and medical staff that result from the application of ML models. Discussion and future research, with ML model limitations, trends, and opportunities, and research study limitations and future general research directions, are given in Section 5. Finally, the conclusions of the study are given in the last section, Section 6.

## 2. Methods

### 2.1. Searched Databases and Review Scope

**Figure 1:**
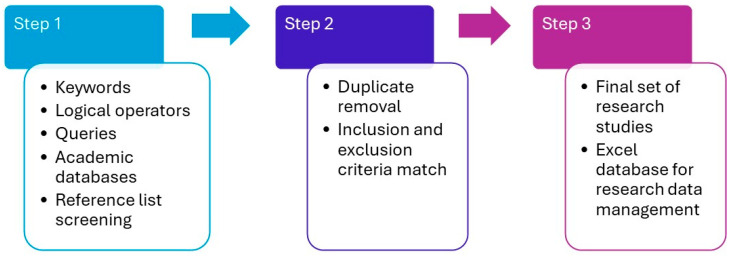
Applied literature review methodology with inclusion/exclusion process (based on proposed literature review methodology [30]).

The studies reviewed in the manuscript were compiled by searching multiple databases, including the Web of Science, Scopus, and PubMed. In order to make a review of research regarding the application of ML models in venous ulcer (VU) wound care, different terms were used. The main keywords on the basis of which the queries were made for searching these databases were “venous ulcer” OR “chronic wound” AND “machine learning” OR “artificial intelligence”. Using the previously mentioned keywords and logical operators (“AND” and “OR”), the search of the Web of Science, Scopus, and PubMed databases generated an initial set of about 1250 potential studies for review, where the combination of keywords “chronic wound” AND “artificial intelligence” yielded the most results. Searching these databases resulted in overlapping results, so duplicates were removed. In addition, some of the reviewed manuscripts were considered based on the screening of reference lists of previously reviewed manuscripts and deeper analysis in order to ensure that VU disease was covered within the research. Moreover, the analysis of review papers with a focus on the application of artificial intelligence in dermatology and medicine [3,23,24,25,26,27,28,29] helped in finding suitable manuscripts. To ensure the scope and relevance of the review, several inclusion and exclusion criteria were applied. Studies were included if full-text versions were available and the publication was in English. Only journal manuscripts and conference proceedings papers were considered for inclusion in this review. Studies were excluded if they did not address venous ulcers or lacked sufficient information regarding ML model development and evaluation. The availability of a detailed description of the clinical study design and the datasets employed was also considered. The search for research studies was performed during the first and second quarters of 2025, and subsequently updated to cover the literature published through August 2025, reporting a total of 79 research studies (65% journal manuscripts and 35% conference proceedings papers) focused on the application of different ML models for considering various aspects of VU wound care. To perform a comprehensive review with respect to different aspects, all relevant data were extracted, synthesized, and stored in an Excel file to facilitate the effective management of research data (Figure 1).

It should be noted that, in general and in this study, systematic literature reviews face several challenges. First, the exclusion of non-English publications may limit the comprehensiveness of the review. Second, the selection of databases can introduce potential bias, as studies not indexed in the chosen databases may be overlooked. Third, comparing ML studies is challenging due to the use of different performance metrics and validation schemes, which can hinder direct comparisons.

### 2.2. Data Extraction Methodology

For each publication, data was extracted using a standardized form which included the manuscript title, authors, date of publication, authors’ country, database, sample size, observation period, type and number of ML models used, data splitting, optimization of ML model hyperparameters, feature engineering, statistics used for assessment of ML model performance, programming language used for ML model development, and primary purpose of the developed ML model. In addition, for each manuscript, an analysis was made with regard to the most important contribution made, the most important observations (insights), observed limitations, as well as advantages and opportunities. This enabled a large number of research papers to be viewed from multiple aspects and facilitated the analysis of research contributions, insights, and findings, as well as future research subjects and directions.

## 3. Analysis of Results

**Figure 2:**
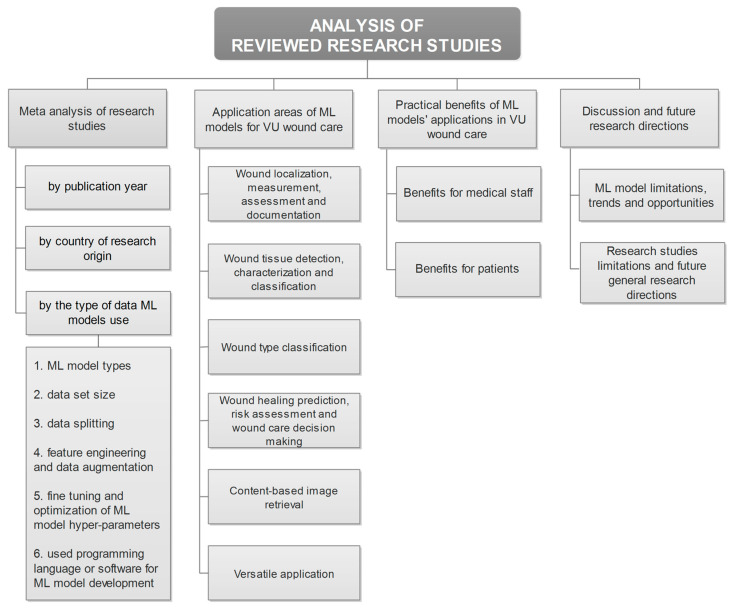
The organization of the conducted research review.

This review presents an overview of the development and application of ML models in VU wound care. The overview of the conducted analyses of reviewed research studies is given in Figure 2. As can be observed, there are four main parts, including meta-analysis of research studies, identified application areas, practical benefits of the application of ML models, and discussion and future research directions.

### 3.1. Meta-Analysis by Publication Year

**Figure 3:**
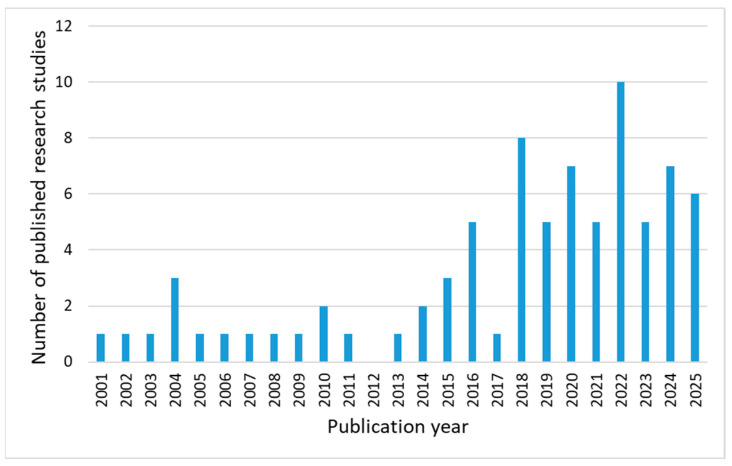
Reviewed studies by publication year.

The first meta-analysis considered the publication year of the research study. As can be observed from Figure 3, the application of ML models for VU wound care covers a research period from 2001 to August 2025. In the first part of that period, until 2015, the number of published research papers is characterized by a level pattern, while the second part, starting from 2015, shows a strong trend of growth in the number of publications. This can be attributed to the previously obtained promising results, advantages, and possibilities, as well as recent advances in ML, which fostered recent research progress in the field. Actually, in the last ten years, i.e., in the period between 2015 and August 2025, six research studies were published on average annually, covering different applications of ML models for VU wound care (Figure 3).

### 3.2. Meta-Analysis by Country of Research Origin

**Figure 4:**
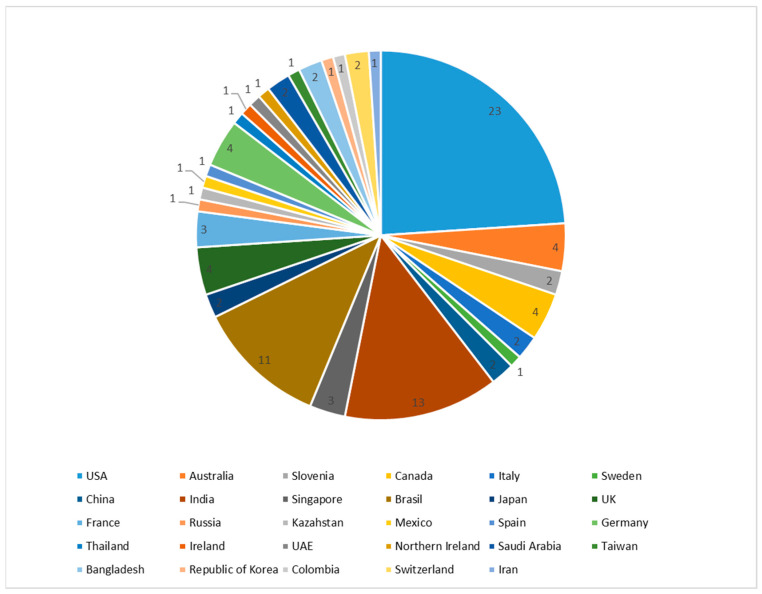
Research studies by country of research origin.

For the purpose of the second analysis, research studies were classified according to the country in which the research was conducted (Figure 4). As can be observed, the research studies were performed around the globe but mainly in the USA, India, and Brazil (48%). Around 21% of the reviewed studies were conducted by authors from Europe, while the rest were the results of authors and researchers from different parts of the world, including Canada, China, Japan, Singapore, Australia, etc. Most of the published research studies are the result of the work of multi-member multidisciplinary teams from one country, although there are also research studies by authors from two or more institutions from different countries.

### 3.3. Meta-Analysis by the Type of Data ML Models Use

**Figure 5:**
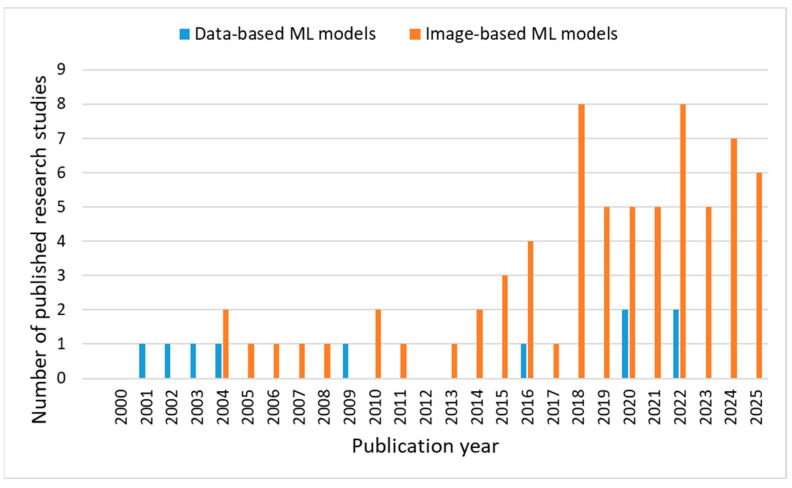
Application of data-based and image-based ML models for VU wound care.

By reviewing the research studies, a clear distinction between the two types of ML models could be made based on the different types of data that ML models use, which ultimately determined the primary application scopes of the developed models. On the one hand, there are ML models that use numerical (discrete and continuous) and categorical (nominal and ordinal) data, referred to in the present research as data-based ML models. On the other hand, a number of recent studies use image data as essential model inputs, referred to in the present research as image-based ML models. As can be observed from Figure 5, around 13% of the reviewed studies are conducted by the application of data-based ML models and 87% by the application of image-based ML models. Although the application of image-based ML models has increased significantly in the previous ten years, data-based ML models are still in use, particularly for specific applications such as VU healing prediction. It has to be noted that two studies were performed by utilizing both wound image and texture features, along with data features such as categorical features for representing wound location [31].

Given that different types of data are used, a separate, more detailed analysis was carried out for both categories of ML models used. To that end, a number of analyses were performed with respect to: ML model types, number of different ML models tested, number of data used for ML model development, data splitting and training-validation-test sets ratios, feature engineering and data augmentation, fine-tuning and optimization of ML model hyperparameters, and programming language or software used for ML model development.

#### 3.3.1. Data-Based ML Models

**Figure 6:**
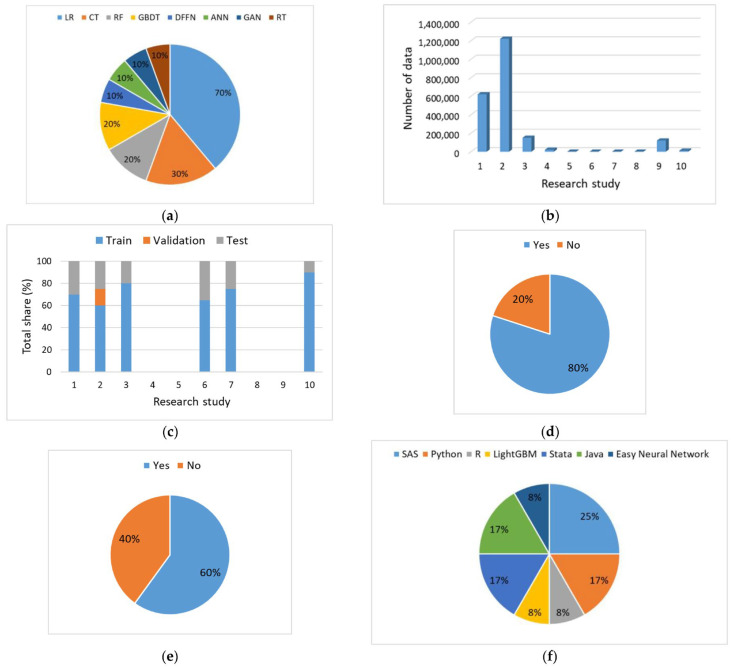
Meta-analysis of data-based ML model development: (**a**) ML model types. (**b**) Number of data used for ML model development. (**c**) Data splitting in ML model development. (**d**) Feature engineering and data augmentation. (**e**) Fine-tuning and optimization of ML model hyperparameters. (**f**) Programming language or software used for ML model development.

Meta-analysis of data-based ML models used in VU wound care, with respect to the previously mentioned aspects, is summarized in Figure 6.

As can be observed from Figure 6a, the most applied ML models are logistic regression (LR), classification tree (CT), random forest (RF), and gradient-boosted decision tree (GBDT) models. On the other hand, the use of artificial neural network (ANN), generative adversarial network (GAN), and regression tree (RT) models is limited. All applied ML models belong to the class of supervised ML models, which are developed using labeled data. Most research studies use a single ML model for solving the given problem; however, the use of two [5,32,33], three [34], or even four ML models [6] is not uncommon. The development of multiple ML models was aimed at finding the best model for solving the specified problem and available data. In these studies, multiple types of data from retrospective and observational studies and electronic medical record (EMR) databases were used for ML model development. As noted by Lee et al. [1], ML models may exhibit different accuracy levels; hence, it is not recommended to rely on a single model in clinical practice. As can be observed from Figure 6b, the number of data used for ML model development ranges from several tens [4], hundreds [16], thousands [35], and even ML data development sets with over a million data points [6].

In ML model development, it is usual practice to use three data sets: training, validation, and test. Initially, a training data set (the majority of data) is the set from which the model learns the underlying data patterns. On the other hand, a validation set is used for validation of the developed model and fine-tuning of ML model hyperparameters, whereas a test data set is used at the end of the model development process for unbiased evaluation and performance check using data that has not been previously used. With regard to the training, validation, and test sets used, as well as their ratios, the analysis of results is given in Figure 6c. In four research studies, data splitting and training-validation-test sets ratios were not explicitly given [9,32,33,36].

Feature engineering, as a basic and very important step in ML model development, refers to the selection, transformation, and/or development of new relevant, more important features that could enhance the ML model development process as well as its predictive capability. Another important pre-processing step is the data augmentation, which refers to the generation of new and diverse data from existing data with the goal of reducing a model’s tendency to overfit, enhancing its performance, and saving required resources available for data collection. In relation to these two key steps in ML model development, a review of the literature shows that the majority of research studies applied feature engineering and data augmentation so as to improve the quality and quantity of data available for ML model development and ensure improved model performance (Figure 6d).

Every ML model is characterized by a set of hyperparameters, which refer to the underlying model topology and training process. Hence, they have a significant effect on ML model training efficiency and its prediction accuracy [37]. Therefore, it is of utmost importance for a given dataset to fine-tune and/or optimize a set of ML model-specific hyperparameters so as to avoid underfitting and overfitting issues. As can be observed from Figure 6e, fine-tuning or optimization of ML model hyperparameters was reported in 60% of studies that used data-based ML models.

As can be observed from Figure 6f, for the purposes of ML model development and analysis, ready-made statistical software packages such as SAS and Stata were most often used.

#### 3.3.2. Image-Based ML Models

**Figure 7:**
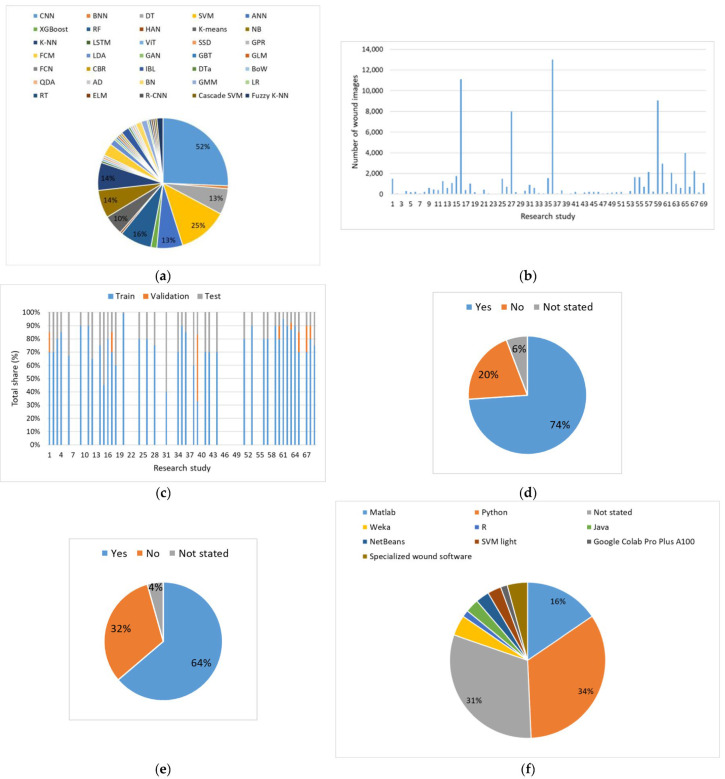
Meta-analysis of image-based ML model development: (**a**) ML model types. (**b**) Number of wound images used for ML model development. (**c**) Data splitting in ML model development. (**d**) Feature engineering and data augmentation. (**e**) Fine-tuning and optimization of ML model hyperparameters. (**f**) Programming language or software used for ML model development.

A meta-analysis of image-based ML models used in VU wound care, with respect to different aspects of ML model development, is summarized in Figure 7.

Unlike data-driven models, where 8 different ML models and algorithms were used, in the work with VU wound images, 35 different supervised, unsupervised, and reinforcement ML paradigms are used (Figure 7a). Approximately half of the research studies are based on the application of convolutional neural network (CNN) models, and in every fourth study, support vector machine (SVM) models are used. The following types of models also have a considerable number of applications: random forest (RF), k-nearest neighbors (KNN), Naïve Bayes (NB), artificial neural network (ANN), decision tree (DT), and the K-means clustering algorithm. A review of the literature reveals that the following types of ML models and algorithms have very limited application: Bayesian neural network (BNN), hierarchical attention network (HAN), long short-term memory (LSTM), visual transformers (ViT), single-shot multibox detector (SSD), Gaussian process regression (GPR), generative adversarial network (GAN), gradient boosted trees (GBTs), general linear model (GLM), fully convolutional network (FCN), case-based reasoning (CBR), decision table (DTa), bag-of-visual-words (BoW), quadratic discriminant analysis (QDA), AdaBoost model (AD), logistic regression (LR), regression tree (RT), extreme learning machine (ELM), region-based CNN (R-CNN), and cascade SVM. Over half of the research studies tested at least two ML models, while some research studies developed and compared multiple ML models. As a prominent example, one can mention research studies where even nine ML models were developed [38,39].

An overview of the number of wound images that were used for the development of ML models is given in Figure 7b. It has to be noted that for a clearer presentation of the results, the data from two research studies were omitted. Namely, the research study of Gupta et al. [40] is based on a wound image database containing over two million wound images, while 469,000 wound images were used by Ramachandram et al. [41]. Leaving these two studies out of consideration, it can be said that the average number of wound images used for ML model development is around 1200. The number of wound images was not explicitly stated in only two studies [42,43].

With regard to the training, validation, and test sets used, as well as their ratios, the analysis of results is given in Figure 7c. As in the case of data-based ML models, approximately 40% of research studies omit precise information regarding data splitting. Only a few studies used three separate data sets for training, testing, and validation, in order to ensure an effective training process and powerful generalization capability of ML models with consistent performances [44,45,46,47].

Development of image-based ML models in most cases is based on feature engineering, data augmentation, and fine-tuning or optimization of hyperparameters (Figure 7d,e). However, feature engineering and data augmentation of image data are somewhat more often applied. Finally, as can be observed from Figure 7f, programming languages, primarily Python and Matlab, and data analysis free ML applications (Weka, SVM light) were mostly used for image-based ML models. For the purpose of solving different aspects of VU wound care, some research studies used specialized wound software such as WoundAide [15], Droice Labs [48], and Tissue Analytics [22], which incorporate multiple ML models for specific purposes. It has to be noted that approximately one-third of the research studies did not specify the programming language or software that was used for ML model development.

A more detailed analysis of ML model development is given within the next section, which refers to the overview of research studies according to a specific application domain, where some more information is given in tabular format.

## 4. Application Areas of ML Models for VU Wound Care

This review involved an analysis of 79 research studies focused on the application of ML models in the field of VU wound care. Within that corpus, 10 studies developed and applied data-based ML models, while 69 studies used image-based ML models. After initial meta-analyses of research studies, an attempt was made to identify the main application areas of ML models for VU wound care with additional important information. In addition, the key advantages and possibilities of ML models for VU wound care, as well as the main benefits that ML models bring to medical workers, patients, and the healthcare system in general, are highlighted.

Based on the review of research studies and the analysis of the set goals, six application areas were identified, where ML models mainly address prediction, classification (binary and multi-class), and decision-oriented tasks.

### 4.1. Wound Localization, Measurement, Assessment, and Documentation

The first application area refers to the use of ML models for wound localization, wound size/area measurement, wound segmentation (boundary detection), comprehensive wound assessment, and wound documentation. Table 1 gives a summary of research studies in this application area with specific information related to ML model development and the conducted study.

### 4.2. Wound Tissue Detection, Characterization, and Classification

Within VU and application of ML, a significant number of research studies focused on wound tissue detection, tissue characterization, and classification of different tissue types (granulation, slough, necrotic, etc.), as wound tissue classification is essential for wound assessment, monitoring, and treatment planning [67,68]. Table 2 gives a summary of research studies within this application area.

### 4.3. Wound Type Classification

The third identified application area refers mainly to the application of ML models for multi-class wound type classification that is essential for wound diagnosis [31] and timely and adequate wound treatment [44]. Table 3 gives a summary of research studies within this application area.

### 4.4. Wound Healing Prediction, Risk Assessment, and Wound Care Decision-Making

Within this application area, a number of research studies applied ML models (both data and image-based) for the prediction of wound healing probability and outcomes, risk stratification, identification of delayed wound healing, wound healing assessment, wound care decision-making, etc. Table 4 gives a summary of research studies within this application area.

### 4.5. Content-Based Image Retrieval

Several research studies focused on developing content-based image retrieval (CBIR) systems for chronic wounds. CBIR systems, given a set of image features, locate, retrieve, and display images similar to the one given as a query [90]. Given that they provide relevant past cases with proven pathology and associated clinical, diagnostic, and other information, they can be of great importance and usefulness for doctors and medical workers [91]. Table 5 gives a summary of research studies within this application area.

### 4.6. Versatile Application

Many research studies predominantly focus on the identification of specific medical conditions, such as wound detection, wound area segmentation, or wound image classification, individually. Those studies may lack the capability to provide holistic recommendations [12]. However, several studies attempted to offer more comprehensive solutions by proposing a unified framework for addressing several tasks at the same time, including wound measurement, segmentation, classification, wound stage classification, infection detection, wound treatment plan recommendation, healing prediction, etc. Table 6 gives a summary of research studies within this application area.

### 4.7. Benefits for Medical Staff and Patients

The development and application of ML models and ML-based systems in VU wound care offers a number of benefits both for medical staff and healthcare systems as well as patients (Table 7).

#### 4.7.1. Benefits for Medical Staff and Healthcare Systems

The use of ML models and ML-based systems and applications can provide consistent, objective, instantaneous measurements and assessment of wound tissues and chronic wounds [22,48], thus reducing inter-observer variability and helping to find proper wound treatment plans [31]. As noted by Howell et al. [48], they have the potential to improve the accuracy and consistency of wound area and wound tissue measurements while improving the efficiency of wound care workflows. Further, the use of ML increases the simplicity of the procedure, reduces computational costs, and improves the diagnosis [59].

The application of ML models in VU wound care not only helps remove human subjectivity but also accelerates wound assessment and clinical practice [1,21,22], even in resource-limited settings [31,44]. This enables a more comprehensive assessment of patients, as well as more efficient work of the medical staff. As noted by Robnik-Šikonja et al. [33], if the wound healing rate is known, the provided information can help to formulate the appropriate management decisions, reduce the cost, and orient resources towards individuals with poor prognosis. Also, there is potential to use ML to detect wounds that may be slow to heal or require prompt medical attention, allowing triage of care while decreasing strain on healthcare resources [48]. Wound status monitoring using automated ML-based methods provides superior performance, including a reduction in treatment-related burdens, minimized cost of care, and an accurate assessment [72]. Proposed automated chronic wound healing systems [31,44] offer cost-effectiveness and aid clinicians in prompt diagnosis and development of suitable treatment plans. However, this necessitates proper technical training for both patients and medical staff.

There exists a lack of decision support for non-expert clinicians who usually provide most wound assessments and care decisions at the point of care (POC) [88]. Existing decision support systems are limited to rubrics or questionnaires that have to be filled out manually to generate decisions [89]. The use of ML-based VU wound care systems with supported mobile phone applications can recommend actionable chronic wound care decisions, including emergent situations [1], and aid a registered nurse and medical staff in deciding what treatment a chronic wound requires, thereby standardizing wound care and wound management guidelines [1,89]. Wound care decisions generated autonomously by an ML-based system could provide the necessary support and aid for non-expert care providers by minimizing uncertainty during wound care decision-making [88] and ultimately improving wound care decision consistency and reducing costs [89]. Avoidance of inconsistent decision-making across the wound care community will promote high-quality wound care and protect chronic wound patients [88]. Prompt and accurate wound assessment, provided by automated ML-based systems, can prevent wound misdiagnoses by care professionals, which could lead to detrimental or irreversible clinical outcomes [1]. The ability to identify patients at high risk of having wounds that will not heal or heal after an abnormal amount of time may facilitate data-driven clinical decision-making to limit complications, improve patient outcomes, and reduce costs of care [6,40]. Also, information on wound healing progress has multiple benefits for clinical decision-making [5].

Within the telemedicine framework, ML-based systems and applications can improve communication between clinicians [22], while multiple expert clinicians can be able to provide correct diagnoses for wound patients [97]. In addition, clinicians in resource-limited settings can quickly identify the types of wounds and seek help from experts accordingly based on the initial wound assessment [31]. Remote monitoring, assessment, and control of wound care ensure clinical effectiveness and minimize the healthcare burden [31,97]. In addition, ML-based wound analysis equipped with mobile devices allows rapid diagnosis and quality treatment, especially for rural or underserved regions with much less accessible resources [31].

With the existence of extensive and comprehensive wound databases [78], development of wound CBIR systems [38], the possibility to provide real-time wound characteristics and treatment recommendations [12,36,95], and the possibility to train medical staff who may have less experience in wound care management [47], ML-based systems and application in VU wound care have multiple significant educational and training values. In addition, the use of explainable machine learning models can help clinicians to understand how the model derives its conclusion [47], thus enhancing education and training opportunities.

ML-based systems and applications can provide standardized key information for all wounds that link to the patient’s eMR [22], eliminating redundancy and improving documentation consistency and completeness. Such apps are reliable and powerful tools for wound documentation, where information is easily accessible and available across multiple healthcare providers. Rather than paper-based wound documentation, electronic wound assessments allow systematic tracking of wound healing progress and minimize errors or incomplete assessments [41]. In addition, such systems could provide information about prognosis as well as linking this information to treatment guidelines, which facilitates patient continuity of care [22]. Because it is computer-based, the costs to update this system should be minimal as medical knowledge and drug development advance [36].

#### 4.7.2. Benefits for Patients

From the aspects of wound care accessibility, patient comfort and safety, there are significant benefits, including remote patient monitoring and consultations with wound care experts in acute and outpatient settings [22], accessibility to wound care experts in rural and underserved areas for rapid diagnosis and quality treatment [31], and access to improved diagnostic and management strategies for patients in distant locations [51]. Patient benefits may also include reduced discomfort for the patient and the doctor’s ability to view the wound remotely, eliminating the unnecessary early removal of the dressing [22]. Furthermore, patients with basic medical knowledge can use the medical advisory service on the computer system, helping them to seek medical care in time [63], and allowing for efficient diagnosis and treatment remotely without attending a clinic [97]. In addition, acceptable wound assessment results can be obtained in an automatic, non-invasive approach, which avoids discomfort to the patient and eliminates the risk of contamination of the wound [55]. Moreover, the use of ML-based applications facilitates patients’ documentation and data management of wound care and communication between patients and medical staff [22]. As noted by Chan et al. [15], the use of ML-based wound imaging systems permits quick wound measurement and assessment, reducing the time of examination of patients as well as the use of multiple complex setups. Finally, benefits also include increased simplicity of procedure [59] and the use of simple equipment such as a smartphone or computer.

The use of ML models and ML-based wound care systems and applications offers significant benefits for earlier detection and intervention with better wound treatment outcomes. Wound assessment in the initial stage using ML models enables medical staff to conduct more accurate and faster detection of high-risk wounds [40], faster referral to specialists [35], better diagnosis, and timely, adequate wound treatment, which helps patients significantly [83] and reduces the recurrence rate [69]. Early, accurate prediction of delayed healing wounds can improve patient care by allowing clinicians to increase the aggressiveness of intervention in patients most at risk [34]. With the use of ML prognostic models, one can select patients who are unlikely to heal for enrolment into a randomized clinical trial of a new therapy [9]. It has also been argued that one can improve patient outcomes with higher efficiency [6,31] and prevent negative patient outcomes [22]. Possibilities of using real-time wound assessment solutions capable of tracking dynamic changes in wound appearance and monitoring healing progress over time [95] would significantly improve patient wound treatment outcomes.

The possibility of monitoring wound progression or deterioration and managing their own wound care under supervision enhances patients’ engagement and adherence in wound care [22]. The development and use of ML-based systems and applications enable patients to perform self-diagnosis and consult a doctor on time [64], improve self-care [1], and provide an estimate of wound healing time to motivate patients to follow up their treatments [4].

In addition to being not expensive for implementation in clinical practice, ML-based systems and applications can improve patient outcomes with higher efficiency and lower wound treatment and healthcare costs for patients [31,72]. Reduced healthcare costs result from saved patient travel time and timely wound care treatments, as well as from maintaining optimal wound care [22] and minimizing repeated and unnecessary clinic visits [97].

The proposed ML-based systems and applications can provide standardization and offer a better quality of treatment for patients [97]; however, their use also enables personalized wound treatments, offering assistance in designing optimized and personalized therapy for each patient [93]. Not only can problematic wounds that require advanced therapies to heal be identified [35], but novel therapies can also be recommended as a replacement for standard therapy [16].

## 5. Discussion and Future Work

This section provides a summary of the general findings from the reviewed articles with respect to limitations, trends, and opportunities of the ML model development process, as well as research study concepts and application methodology.

### 5.1. ML Model Limitations, Trends, and Opportunities

#### 5.1.1. Limitations

Although the vast majority of research studies reported successful application of ML models, their generalization capability, predictive, and classification power may be limited mainly due to small datasets obtained from a single healthcare center [5,15,21,60] and imbalanced data sets [8,60]. Also, as noted by Huang et al. [82], fine-tuning of ML models with additional data obtained under more diverse conditions is required for prospective use. The limitations of ML models result from the diversity of available data and the use of biased data sets [58]. In some research studies, there was a strong prevalence of wound data of certain ethnic groups’ skin tone, such as Caucasian patients [54] or Japanese patients [50]. In general, many ML models lack transparency and are usually considered as black boxes [79], which provide certain results without being able to understand the background of the decision-making process. As noted by Frasca et al. [24], the lack of interpretability and explainability of ML models is the main reason that limits the wider adoption of ML-based solutions in medicine, as it raises legal and ethical concerns. Overfitting and underfitting problems were not reported, probably given that the majority of research studies applied ML hyperparameter tuning and/or cross-validation, or at least attempted some trial-and-error approach [8]. Related to the application of image-based ML, VU image processing limitations and challenges, such as varying lighting conditions, image quality, background with complex textures, image acquisition protocols, etc., are to be noted. In the extreme case, one or more of these issues can lead to nonskin backgrounds being misjudged as skin, which may in turn result in some nonskin backgrounds being misjudged as wounds [98]. Training deep learning models, particularly suitable for VU image data, is resource-intensive and requires considerable computational power. For reducing computational costs, fine-tuning of image-based ML models is essential [44].

#### 5.1.2. Trends and Opportunities

Trends and opportunities provide new approaches for improving ML model efficiency, transparency, performance, and application scope, while also contributing to the overall enhancement of VU wound care. Based on the literature review, one can highlight integrative and multimodal approaches, real-time processing, and ML model architecture as main research topics.

Integrative and multimodal approaches, aimed at improving decision-making, accuracy, and generalization capabilities of ML models, refer to (1) the use of combined multiple data modalities (wound images, wound-level characteristics, wound location, patient demographics, comorbidities, etc.) [31,44], (2) the integration of classical (such as Cox proportional hazards model) and ML models [40], (3) the development of multi-task ML models [7,83], and (4) the fusion of different ML models (such as CNN for wound segmentation, SVM for classification and GP regression for healing prediction) [96].

The possibilities of real-time wound data processing include the development of mobile/smartphone, edge computing, and telemedicine applications, web service prototypes [31,54,60,72,78,82,88,97], real-time tracking and monitoring of wounds, analysis of wound characteristics [22,95], integration of EMR records [36], and prediction of the probability of healing of chronic wounds or PWAT score [6,21]. In an attempt to develop ML models for successful data mining, pattern identification, knowledge discovery, and modelling of relationships, a comprehensive analysis of the ML model development workflow is crucial [99]. It refers to data analysis and preprocessing, feature extraction and selection, model selection, training algorithms, and evaluation metrics. A critical aspect of this workflow relates to providing high-quality input data so as to enable reliable usage of developed ML models. In that regard, image enhancement is a key preprocessing step for contrast improvement, noise reduction, and preservation of relevant structures. In the subsequent analysis stage, these enhanced images facilitate accurate feature extraction, with such features then being used as input to ML models, either as classifiers or regressors. Some of the recent studies in VU wound care proposed a well-designed framework with careful consideration of all main issues in the design of ML architecture, as well as the application of optimization methods and techniques for ML model improvement [44]. Moreover, explainable ML models for analysing wound images are proposed for easier integration and acceptance of ML-based wound applications in healthcare systems [47]. Likewise, image-based research studies, in particular, leverage pre-trained CNN models (InceptionV3, Resnet50, VGG16, InceptionResnetV2, YOLOv3, AlexNet, U-Net, Segnet, MobileNet, and EfficientNetB0) and transfer learning techniques to propose innovative solutions for VU wound care, even with a limited set of wound images [46,50,62,83].

Image quality remains critical for ML model performance. High-quality wound images with enhanced contrast, reduced noise, and improved visibility of relevant features provide richer and more reliable information for ML models. These enhancements improve feature discrimination, reduce spurious features, and enable revealing subtle, meaningful patterns and relevant details, thereby leading to improved segmentation, measurement, classification, and prediction accuracy in wound care tasks. It has been proven that even advanced deep learning architectures benefit from image preprocessing and enhancement because clearer inputs reduce ambiguity, facilitate more effective feature learning, and improve model training effectiveness and generalization [66]. Given their significance for ML model performance and wound diagnostic reliability, several enhancement frameworks have been proposed as critical preprocessing steps to improve the visual quality of medical images, particularly relevant to diagnostic reliability [100,101,102]. Such enhancement strategies are supported by both theoretical and experimental validations and have been shown to improve the reliability of downstream analysis tasks, including segmentation, measurement, and classification, by providing clearer and more discriminative inputs to ML models.

Finally, one should mention some recent innovative model architecture approaches, such as (1) the application of a conditional generative adversarial network (cGAN) with an aim to assist in proposing proper chronic wound treatment plan by understanding the border segmentation and the wound tissue classification visually [79], (2) the application of data-efficient image transformers (DeiT) and a custom vision transformer, as a new techniques for image classification, that outperformed pre-trained CNN models for solving multi-classification task of automatic chronic venous disease [64], (3) the application of bag-of-visual-words (BoVW) for development of content-based image retrieval system for dermatological ulcer images [38], and (4) the application of region-based CNNs (R-CNNs) to detect and differentiate wounds and classify their tissues with promising results, providing an efficient and convenient tool for doctors to raise the bar for therapeutic healing of wounds [95].

### 5.2. Research Study Limitations and Future General Research Directions

#### 5.2.1. Limitations

First of all, the retrospective nature of conducted research studies limits causal inference, introduces risk of covariate bias and potential confounders, and limits prospective validation of ML models’ predictions. As noted by Cho et al. [5], ideally, predictions made would have to be validated prospectively. In some studies, clinical interventions were not used as covariates in the models [6], while in others, the analysis of prognostic indices on clinical interventions was omitted [40], thus failing the evaluation of the effectiveness of wound treatment strategies [3]. Prospective application may also require some fine-tuning of ML models with additional data, particularly those captured under more diverse imaging conditions in different settings [82]. Limited integration with clinical workflow and electronic health records for ease of use is another issue to be resolved in future study concepts and proposed methodologies [47]. Many image-based ML models were developed using wound sets gathered from a single institution under the same conditions and wound image acquisition protocol. This, however, may result in less reliable ML models that demonstrate lower prediction capability when using wound images from multiple healthcare centers [1], since there is a lack of standardized tools for wound image acquisition [103]. On the other hand, the quality and consistency of clinical documentation vary across many practitioners and centers [35], affecting retrospective studies that use clinical data from multiple sources and decision-making based on ML model predictions. The limited available image datasets with associated clinical notes (decisions and measurements) used in research studies may not be sufficient to demonstrate the generalizability of the ML models [88]. The lack of standardization in wound assessment protocols poses a significant challenge to the wider application scope of research studies. Inconsistencies, subjective assessments, and possible errors within and across institutions and among specialists and nurses make it difficult to obtain reliable data [95]. Although many ML-based research studies in VU wound care demonstrate promising results, a notable gap remains in clinical practice, due to the hardware limitations, privacy concerns, and integration challenges with existing healthcare systems [95].

#### 5.2.2. Future General Research Directions

Based on the literature review, one can identify the following future general research subjects and directions in VU wound care research for all identified application areas: (1) the need to collect more comprehensive wound data sets with diverse features, (2) the focus on clinical applications and integration with clinical workflow, and (3) a more comprehensive clinical validation.

In order to enhance ML model robustness and its applicability for VU wound care, further research should consider larger wound data sets [2,45,47,83,93], wound data sets from multiple hospitals [1], multi-ethnic populations and skin tones [47,58], and a wider pool of participants in a variety of settings [8]. In addition, further research should also include wound image data sets introduced with additional variations/noise (lighting, angles, distances) [54,59,81], the use of a more comprehensive set of features [5,82,92], the perception of the treatments and interactions of time and other features for an even more accurate prediction [6,40,48], the use of combined data, i.e., wound image and non-image features [81], leveraging textual features derived from expert comments to improve the accuracy of decisions [89], and multiple wound data sampling [4,5]. It should be noted that medical image enhancement plays a fundamental role as a prerequisite for the reliable use of image-based ML models for segmentation, classification, measurement, diagnosis, and prediction, particularly in clinical scenarios where images are affected by contrast degradation, noise, and acquisition variability. Recently proposed degradation- and contrast-adaptive methods demonstrate effective noise suppression and significant medical image quality improvement and robustness, while preserving clinically relevant information and structure [104,105,106]. Incorporating such enhancement strategies is essential for improving the generalizability and clinical applicability of ML-based systems, especially in real-world scenarios where image quality cannot be guaranteed.

Clinical application fields refer to the possibility of developing automated ML-based systems for accurate diagnoses [1], wound healing [34,54], wound assessment [67], wound type classification [31,81], wound tissue segmentation [41], classifying and quantifying tissue types and ulcer stages [12], wound status monitoring [72], wound segmentation [54], providing real time wound characteristics and treatment recommendation [12,36,95], telemedicine supported systems [22,72,78,97], and patient self-diagnosis solutions [64].

For evaluating the effectiveness and accuracy of developed ML models and ML-based systems, a more comprehensive clinical validation is needed. This includes the necessity of using a separate external cohort for external validation [47], involving medical experts in verifying and instilling confidence in the obtained results [12], validation with larger and more comprehensive data sets [44,55], prospective validation of results prior to implementation [5], monitoring performance on recent cases to guard against changes in practices, patient populations, and other factors degrading performance [34], comparing the performance of ML models with human counterparts and evaluating the effectiveness and accuracy of each [1]. As noted by Ramachandram et al. [41], verification, validation, and continued monitoring are the core of the deployment of ML models in clinical practice.

More comprehensive data collection covering broader patient demographics, along with consistent clinical evaluation and validation [107], is essential for real-world clinical integration of ML models, ensuring safety and generalizability. Future research should also focus on enhancing ML model interpretability and explainability to build clinician trust and support decision-making in practice, as these remain key barriers to adoption by healthcare professionals [28]. Integrating medical domain knowledge into ML systems can further improve interpretability and alignment with clinical workflows and guidelines, facilitating practical implementation [108]. Furthermore, it has been observed that combining both retrospective and prospective data, along with standardizing protocols for ML model development and evaluation, further supports clinical integration [65]. Finally, successful implementation in real-world clinical settings requires addressing existing significant challenges related to privacy concerns, regulatory compliance, interoperability, and integration challenges with existing healthcare systems [95].

## 6. Conclusions

This study systematically reviewed 79 research studies published between 2001 and August 2025 that focus on the application of ML models in venous ulcer wound care. The analysis revealed significant progress in this field over the past decade, reflecting the increasing awareness of the capabilities of machine learning models to tackle the complex challenges in venous ulcer wound care. Across six identified main domains, both data-based and image-based ML models showed promising results, with image-based ML models increasingly dominating recent research due to advances in deep learning architectures and the availability of larger wound image datasets. Classical, data-based ML models have a limited application field and are almost exclusively used within wound healing prediction, risk assessment, and wound care decisions.

The practical benefits of ML applications in venous ulcer wound care are substantial and multifaceted. For healthcare providers and systems, ML models offer several important benefits, such as accuracy and consistency of wound assessments, saving time and resources, supporting clinical decision-making, enabling remote care, enhancing training opportunities, and helping standardize documentation and monitoring. On the other hand, for patients, the application of ML-based venous ulcer wound care solutions can lead to better access to care, earlier detection and treatment of wounds, more personalized care plans, improved treatment outcomes, greater involvement in their own care, and potentially lower healthcare costs.

Although the results reported are promising, there are still several issues to be addressed. First of all, many studies developed their ML models using small, unbalanced, or non-representative data sets. In addition to the lack of standardization in wound image acquisition and assessment, the protocol development of cross-institutional ML models of higher generalizability remains a challenge. Also, most research studies were retrospective, making it harder to provide causal inference, strong conclusions, or validate results in the real world. One should also point out that the limited interpretability of ML models may be a key factor for their acceptance in clinical practice. Finally, the integration of ML-based applications in current healthcare systems remains a challenge as well. Therefore, future research should prioritize the use of larger, more diverse datasets to develop easily integrative, flexible, and upgradable applications and systems, while ensuring their rigorous clinical validation. Integrative and multimodal approaches, development of multi-task ML-based frameworks, the adoption of novel ML architectures, as well as the application of explainable AI models, are emerging as future research directions.

In conclusion, the results from reviewed studies showed that ML models have strong potential for multifaceted improvement of venous ulcer wound care. However, practical clinical realization will require continued collaboration among clinicians, data scientists, and healthcare administrators to address existing limitations, adopt standardized practices, and ensure robust validation and full integration into clinical practice.

## Figures and Tables

**Table 1:** Summary of research studies within wound localization, measurement, assessment, and documentation.

Study	Wound Etiology	Dataset	Observation Period/Number of Patients	Publicly Available Data	Task	ML Models	Model Performance Measures	Software
[15]	VU	222 images	21 months/ 52	No	WM	ML-based wound imaging device (WoundAide)	N/A	N/A
[21]	VU and other CW	612 images	31 months/ 474	Yes	WA	CNN, LASSO regression	ρ	Python
[49]	VU and other CW	446 images	3 months/ 240	No	WS, WM	CNN	JC, DC, precision, recall, RPE	Python
[50]	VU, DFU	396 images	Not stated/ 440	No	WS	CNN	AUC, DC, MCC, accuracy	Python
[51]	VU, DFU, PU	1010 images (2 sources)	Not stated/ Not stated	Partially	WL	CNN (YOLOv3, SSD)	Precision, recall, DC, IoU, MAP	Python
[41]	VU, AU, PU	469,000 images	Swift wound database	No	WS	CNN	mIoU, precision, recall, DC, SE, SP	Not stated
[22]	VU and other CW	427 images	11 months/ 290	No	WD	ML-based wound imaging software (Tissue Analytics)	N/A	N/A
[42]	VU and other CW	Not stated	Medetec database	Yes	WS	CNN	CNR, accuracy rate	Not stated
[52]	VU and other CW	96 images	3 months/ 8	No	WS	SVM	PE	SVM^light^
[53]	VU and other CW	73 images	Not stated/ Not stated	No	WS	SVM	PE	SVM^light^
[54]	VU and other CW	1564 images	21 months/ 474	Yes	WS	CNN (U-Net CNN, PSPNet)	DC, precision, recall	Python
[55]	VU and other CW	77 images	Medetec wound database	Yes	WS	k-means	Accuracy, PPV, SE	Not stated
[56]	VU, AU	217 images	Not stated/ Not stated	Yes	WS	k-NN, DT, RF, ANN, NB, BN, IBL	SE, SP, MAE, κ	Not stated
[57]	VU and other CW	68 images (2 sources)	Not stated/ 42	Partially	WS	k-means, FCM, GMM, RF	SE, SP, DC, JC	Not stated
[58]	VU, DFU, PU	105 images (2 sources)	Not stated/ 64	Partially	WS	k-means, FCM	Accuracy, PPV, Fleiss’ kappa	Matlab
[59]	VU	33 images	Not stated/ 8	No	WS	k-NN, SVM, RF, DT, ANN, NB, ELM	Accuracy, recall, precision, DC	R
[60]	VU, DFU, PU, SW	1639 images	Not stated/ Not stated	No	WA	CNN (ResNet50, EfficientNetB0)	Accuracy, DC, SE, SP	Python
[61]	VU, DFU, PU, SW	1639 images	Not stated/ Not stated	No	WA	CNN	Accuracy, DC, SE, SP	Python
[62]	VU and other CW	188 images	Not stated/ Not stated	Yes	WS	CNN (YoloV4, U-net and MobileNetV2)	Precision, recall, DC	Python
[48]	VU and other CW	199 images	Not stated/ 199	Yes	WA	ML-based wound software (Droice Labs)	N/A	N/A
[63]	VU	221 images	Not stated/ 217	Yes	WA	SVM, k-means	Accuracy, κ, DC, ROC, AUC	Matlab
[64]	VU	11,118 images	Not stated/ Not stated	No	WA	CNN, ViT	Precision, recall, DC	Python
[14]	VU	1770 images	Not stated/ 150	No	WA	k-NN	SE, SP, accuracy	Matlab
[65]	AW, VU and other CW	4000 images	Not stated/ 42	No	WA	CNN (ResNet50, ResNet101)	DC, JC, recall	Python
[66]	AW, VU and other CW	2230 images	WTS, DFUC, FuSeg and STANDUP datasets	Partially	WS	CNN (dual attention U-Net model)	DC, JC	Python

VU—venous ulcer; AW—acute wound; CW—chronic wound; DFU—diabetic foot ulcer; PU—pressure ulcer; AU—arterial ulcer; SW—surgical wound; WM—wound measurement; WA—wound assessment; WS—wound segmentation; WL—wound localization; WD—wound documentation; SSD—single-shot multibox detector; ρ—Spearman’s rank correlation coefficient; RPE—relative percentage error; AUC—area under the receiver operating characteristic curve; MCC—Matthew’s correlation coefficient; JC—Jaccard coefficient; DC—Dice coefficient; IoU—intersection over union; MAP—mean average precision; mIoU—mean intersection over union; CNR—contrast-to-noise ratio; PPV—positive predictive value; MAE—mean absolute error; κ—Cohen’s kappa coefficient; SE—sensitivity; SP—specificity.

**Table 2:** Summary of research studies within wound tissue detection and classification.

Study	Wound Etiology	Dataset	Observation Period/Number of Patients	Publicly Available Data	Task	ML Models	Model Performance Measures	Software
[67]	VU and other CW	74 images	Medetec wound database	Yes	WTC	SVM, NB	κ	Not stated
[69]	VU	1250 images	Not stated/ Not stated	Yes	WTC	CNN	Accuracy, SP, SE	Matlab
[70]	VU and other CW	Several hundreds of images	Not stated/ Not stated	No	WTC	SVM, k-means, k-NN, fuzzy k-NN	SE, SP, accuracy, κ	Not stated
[11]	VU	75 images	Not stated/ Not stated	No	WTC	SVM	Not stated	Not stated
[13]	VU	33 histopathological images	Not stated/ Not stated	No	WC	SVM, k-NN	SE, SP, accuracy	NetBeans
[71]	VU	20 images	Not stated/ Not stated	No	WTC	Cascade SVM	Accuracy	Matlab
[72]	VU and other CW	203 images	Medetec wound database	Yes	WTC	FCM, LDA, DT, NB, RF	Accuracy	Matlab
[73]	VU and other CW	350 images	Not stated/ Not stated	No	WTC	CNN (AlexNet), SVM	Accuracy	Matlab
[46]	VU and other CW	30 images	Not stated/ Not stated	No	WTC	CNN (SegNet, U-net, FCN-32s and FCN-8s)	Accuracy, DC, SE, SP	Python
[43]	VU	Not stated	Not stated/ Not stated	No	WTC	CBR	Accuracy, κ	Not stated
[74]	VU	172 images	Not stated/ Not stated	No	WTC	NB, ANN, DT, k-NN	SE, SP, AUC	Weka
[75]	VU, AU	215 images	Not stated/ 63	No	WTC	RF, NB, IBL, ANN, DTa	Accuracy, AUC, precision × recall graphs	Not stated
[76]	VU	318 images	Not stated/ Not stated	No	WTC	k-NN	Accuracy	Not stated
[39]	VU, AU	217 images	Not stated/ Not stated	Yes	WTC	RT, RF, NB, BN, IBL, ANN, SVM, CNN (InceptionV3, ResNet)	AUC, κ, MAE, SE, SP, DC	Weka
[77]	VU and other CW	905 images	Not stated/ Not stated	No	WTC	SVM	Accuracy	Not stated
[78]	VU and other CW	Several hundreds of images	Not stated/ Not stated	No	WTC	SVM, k-means, k-NN, fuzzy k-NN	SE, SP, accuracy, κ	Not stated
[79]	VU and other CW	13,000 images	eKare Inc. wound database	No	WTC	GAN, CNN (U-Net, PSPNet)	MSE, DC	Python
[80]	VU, DFU, PU	147 images	DFU, Medetec wound databases	Yes	WTC	CNN (VGG16, ResNet50, DenseNet201, EfficientNetB7, MobileNetV2, InceptionV3, NASNetMobile, and Xception)	Precision, recall, specificity, DC, IoU, MCC, AUC	Python

WC—wound characterization, WTC—wound tissue classification.

**Table 3:** Summary of research studies within wound type classification.

Study	Wound Etiology	Dataset	Observation Period/Number of Patients	Publicly Available Data	Task	ML Models	Model Performance Measures	Software
[44]	VU, PU, SU, DFU	1484 images	Medetec and AZH wound datasets	Yes	WTC	CNN	Accuracy, precision, recall, DC	Google Colab Pro Plus A100
[81]	VU	300 images	Not stated/ Not stated	No	WTC	CNN (VGG-19)	Accuracy, precision, recall	Python
[31]	VU, PU, SU, DFU	1088 images	Medetec and AZH wound datasets	Yes	WTC	CNN, ANN, LSTM	Accuracy, precision, recall, DC	Python
[45]	VU, PU, SU, DFU	400 images	Not stated/ 400	Yes	WTC	CNN (AlexNet), ANN	Accuracy, precision, recall, DC	Matlab
[82]	VU and other CW	2149 images	Not stated/ 1429	No	WTC	CNN	Accuracy, SE, SP, AUC	Not stated
[1]	VU and other CW	9077 images	222 months/ Not stated	No	WTC	CNN (VGG-16, VGG-19, EfficientNet-B0, EfficientNet-B5, RepVGG-A0, and RepVGG-B0)	Accuracy	Not stated
[83]	VU and other CW	256 images	Medetec and additional wound database	Partially	WTC	CNN (U-net)	Accuracy, DC, precision, recall	Not stated
[84]	VU, AU	990 images	Not stated/ Not stated	No	WTC	CNN (The Xception)	Accuracy, precision, specificity, recall, F1-score	Python
[85]	VU, AU	607 images	Not stated/ 72	No	WTC	CNN (ResNet50, ResNeXt50, ConvNeXt, EfficientNetB2, EfficientNetV2)	Accuracy, precision, recall, F1-score	Python
[86]	VU, PU, DFU, SU	730 images	AZH wound dataset	Yes	WTC	Multi-modal approach: CNN (Xception) + GMRNN	Accuracy, precision, recall, F1-score, specificity	Python
[87]	VU, PU, DFU, TU	1095 images	Medetec and AZH wound datasets	Yes	WTC	CNN (Eff-ReLU-Net)	Accuracy, recall, precision, F1-score, ROC curve	Python

SU—surgical ulcer; WTC—wound type classification, AU—arterial ulcer; TU—toe ulcer; GMRNN—Gaussian Mixture Recurrent Neural Network.

**Table 4:** Summary of research studies within wound healing prediction, risk assessment, and wound care decision-making.

Study	Wound Etiology	Dataset	Observation Period/Number of Patients	Publicly Available Data	Task	ML Models	Model Performance Measures	Software
[88]	VU, PU, SU, DFU, AU	2056 images	Not stated/ Not stated	No	WCD	DT, RF, SVM, XGboost	DC, AUC	Not stated
[5]	VU and other CW	620,356	57 months/ 261,398	No	WHP	LR, CT	AUC, AIC	SAS
[6]	VU and other CW	1,220,576	Not stated/ 461,293	No	WHP	LR, RF, GBDT, DNN	AUC, accuracy, SE, SP, PPV, NPV, DC	Python, LightGBM
[34]	VU and other CW	150,277	60 months/ 53,354	No	DWH	LR, RF, GBDT	AUC, Brier score	R
[8]	VU	64 images	12 weeks/ 56	Yes	WHP	BNN	AUC, SE, SP	Matlab
[9]	VU	Not stated	13 years/ 20,793	No	WHP	LR	AUC, Brier score, calibration, discrimination	Stata, SAS
[33]	VU and other CW	300	More than 10 years/ 214	No	WHP	RT, CT	MSE, MAE, gain ratio	Java
[32]	VU and other CW	300	More than 10 years/ 214	No	WHP	RT, CT	RE, accuracy	Java
[35]	VU	10,942	9 years/ Not stated	No	WRS	LR	ROC, HCI	SAS
[40]	VU, PU, DFU, AU	2,151,185 images	Not stated/ 98,407	No	WHP	CNN, CPHM	mIOU, HCI, ROC	Python
[89]	VU, DFU, AU	205 images	Not stated/ Not stated	No	WCD	DT, SVM, ANN, XGBoost, RF, HAN	Precision, recall, DC	Python
[16]	VU	275	36 months/ 325	No	WHP	ANN	Accuracy	Easy Neural Network
[4]	VU and other CW	60	Not stated/ 60	No	WHP	GAN	Accuracy, AUC	Python
[36]	VU, DFU	>120,000	8 months/ 1506	No	WHP	LR	odds ratio	Stata
[2]	VU and other CW	377 images	Medetec wound database	Yes	WHA	GBT, NB, CNN, GLM, RF, DT, SVM	Accuracy, SE, PPV, DC, AUC	Matlab

WCD—wound care decision; WHP—wound healing prediction; DWH—delayed wound healing; WRS—wound risk stratification; WHA—wound healing assessment; PPV—positive predictive value; NPV—negative predictive value; HCI—Harrell’s concordance index (C-index); CPHM—Cox proportional hazards models.

**Table 5:** Summary of research studies within wound CBIR systems.

Study	Wound Etiology	Dataset	Observation Period/Number of Patients	Publicly Available Data	Task	ML Models	Model Performance Measures	Software
[92]	VU, AU	215 images	Not stated/ 63	No	CBIR	k-NN	precision × recall graphs	Java
[38]	VU, AU	217 images	Not stated/ 217	No	CBIR	k-NN, SVM, DT, RF, ANN, AdaBoost, NB, QDA	Precision, recall, accuracy, DC	Python
[93]	VU and other CW	172 images	Not stated/ Not stated	No	CBIR	GMM, k-NN, LR	Precision, AUC	Weka, Java, NetBeans
[94]	VU, AU	186 images	Not stated/ Not stated	No	CBIR	RF	DC, precision × recall graphs	Not stated

**Table 6:** Summary of research studies with versatile applications.

Study	Wound Etiology	Dataset	Observation Period/Number of Patients	Publicly Available Data	Task	ML Models	Model Performance Measures	Software
[47]	VU and other CW	2957 images	48 months/ Not stated	No	WM, WS, WTC	CNN (DenseNet, MobileNet, ResNet, DeepLab, FPN, U-Net)	Accuracy, DC, AUC	Not stated
[95]	VU and other CW	726 images	Not stated/ Not stated	No	WM, WS, WTC	R-CNN, k-means	Precision, recall, DC, AUC, PE	Not stated
[12]	VU	1500 images	Not stated/ 150	No	WTC, WSP, TPR	CNN	SE, SP, precision, recall, DC	Python
[96]	VU and other CW	8000 images	NYU wound database	No	WS, ID, HP	CNN (ConvNet), SVM, GP	mIoU, accuracy, recall, DC, AUC, MAE	Not stated
[7]	VU and other CW	726 images	Not stated/ Not stated	No	WM, WS, WTC	CNN (InceptionV3, Resnet50, VGG16, InceptionResnetV2)	RE, accuracy, DC	Matlab
[97]	VU and other CW	60 images	Medetec and additional wound database	Partially	WTC, WSP, TF	LDA, DT, NB	Accuracy, PE	Not stated

WSP—wound stage prediction, TPR—treatment plan recommendation, ID—infection detection, HP—healing prediction, TF—telemedicine framework.

**Table 7:** Benefits of the development and application of ML models in VU wound care.

Medical Staff and Healthcare Systems	Patients
1. Improved assessment accuracy and objectivity 2. Time, resources, and cost efficiency 3. Enhanced decision support 4. Remote wound care capabilities 5. Improved training and education possibilities 6. Enhanced documentation and monitoring	1. Improved access to wound care, patient comfort, and safety 2. Earlier detection and intervention with better treatment outcomes 3. Enhanced patient engagement and adherence 4. Reduced healthcare costs for patients 5. Personalized wound care

## Data Availability

No new data were created or analysed in this study. Data sharing is not applicable to this article.

## Abbreviations

The following abbreviations are used in this manuscript:

AD	AdaBoost model
AI	Artificial intelligence
ANN	Artificial neural network
AU	Arterial ulcer
AUC	Area under the receiver operating characteristic curve
AW	Acute wounds
BNN	Bayesian neural network
BoVW	Bag-of-visual-words
CBIR	Content-based image retrieval
CBR	Case-based reasoning
cGAN	Conditional generative adversarial network
CNN	Convolutional neural network
CNR	Contrast-to-noise ratio
CPHM	Cox proportional hazards models
CT	Classification tree
CW	Chronic wounds
DC	Dice coefficient
DeiT	Data-efficient image transformers
DFU	Diabetic foot ulcer
DT	Decision tree
DTa	Decision table
DWH	Delayed wound healing
ELM	Extreme learning machine
EMR	Electronic medical record
FCN	Fully convolutional network
GAN	Generative adversarial network
GBDT	Gradient-boosted decision tree
GBT	Gradient boosted trees
GLM	General linear model
GMRNN	Gaussian mixture recurrent neural network
GPR	Gaussian process regression
HAN	Hierarchical attention network
HCI	Harrell’s concordance index (C-index)
HP	Healing prediction
ID	Infection detection
IoU	Intersection over union
JC	Jaccard coefficient
KNN	K-nearest neighbors
LR	Logistic regression
LSTM	Long short-term memory
MAE	Mean absolute error
MAP	Mean average precision
MCC	Matthew’s correlation coefficient
mIoU	Mean intersection over union
ML	Machine learning
NB	Naïve Bayes
NPV	Negative predictive value
POC	Point of care
PPV	Positive predictive value
PU	Pressure ulcer
QDA	Quadratic discriminant analysis
R-CNN	Region-based CNN
RE	Relative error
RF	Random forest
RPE	Relative percentage error
RT	Regression tree
SE	Sensitivity
SP	Specificity
SSD	Single-shot multibox detector
SU	Surgical ulcer
SVM	Support vector machine
SW	Surgical wounds
TF	Telemedicine framework
TPR	Treatment plan recommendation
TU	Toe ulcer
ViT	Visual transformers
VU	Venous ulcers
WA	Wound assessment
WC	Wound characterization
WCD	Wound care decision
WD	Wound documentation
WHA	Wound healing assessment
WHP	Wound healing prediction
WL	Wound localization
WM	Wound measurement
WRS	Wound risk stratification
WS	Wound segmentation
WSP	Wound stage prediction
WTC	Wound tissue classification
WTC	Wound type classification

## References

[1] Lee J.W., You H.J., Cha J.H., Lee T.Y., Kim D.W. (2024). VGG19 demonstrates the highest accuracy rate in a nine-class wound classification task among various deep learning networks: A pilot study. Wounds.

[2] Khalil A., Elmogy M., Ghazal M., Burns C., El-Baz A.A. (2019). Chronic wound healing assessment system based on different features modalities and non-negative matrix factorization (NMF) feature reduction. IEEE Access.

[3] Le D.T.P., Pham T.D. (2023). Unveiling the role of artificial intelligence for wound assessment and wound healing prediction. Explor. Med..

[4] Foomani F.H., Anisuzzaman D.M., Niezgoda J., Niezgoda J., Guns W., Gopalakrishnan S., Yu Z. (2022). Synthesizing time-series wound prognosis factors from electronic medical records using generative adversarial networks. J. Biomed. Inform..

[5] Cho S.K., Mattke S., Gordon H., Sheridan M., Ennis W. (2020). Development of a model to predict healing of chronic wounds within 12 weeks. Adv. Wound Care.

[6] Berezo M., Budman J., Deutscher D., Hess C.T., Smith K., Hayes D. (2022). Predicting chronic wound healing time using machine learning. Adv. Wound Care.

[7] Reifs D., Casanova-Lozano L., Reig-Bolaño R., Grau-Carrion S. (2023). Clinical validation of computer vision and artificial intelligence algorithms for wound measurement and tissue classification in wound care. Inform. Med. Unlocked.

[8] Ngo Q.C., Ogrin R., Kumar D.K. (2022). Computerised prediction of healing for venous leg ulcers. Sci. Rep..

[9] Margolis D.J., Allen-Taylor L., Hoffstad O., Berlin J.A. (2004). The accuracy of venous leg ulcer prognostic models in a wound care system. Wound Repair Regen..

[10] Salomé G.M., Ferreira L.M. (2018). The impact of decongestive physical therapy and elastic bandaging on the control of pain in patients with venous ulcers. Rev. Colégio Bras. Cir..

[11] Anusha D.N., Bhavani R.R. (2016). Classification of varicose ulcer tissue images. Adv. Nat. Appl. Sci..

[12] Rajathi V., Chinnasamy A., Selvakumari P. (2024). DUTC Net: A novel deep ulcer tissue classification network with stage prediction and treatment plan recommendation. Biomed. Signal Process. Control.

[13] Ajitha K. (2020). SVM vs. KNN for Classification of histopathological images of varicose ulcer. Adv. Eng. Int. J..

[14] Bhavani R.R., Wiselin Jiji G. (2017). Image registration for varicose ulcer classification using KNN classifier. Int. J. Comput. Appl..

[15] Chan K.S., Liang S., Cho Y.T., Chan Y.M., Tan A.H.M., Muthuveerappa S., Lai T.P., Goh C.C., Joseph A., Hong Q. (2022). Clinical validation of a machine-learning-based handheld 3-dimensional infrared wound imaging device in venous leg ulcers. Int. Wound J..

[16] Taylor R.J., Taylor A.D., Smyth J.V. (2002). Using an artificial neural network to predict healing times and risk factors for venous leg ulcers. J. Wound Care.

[17] Bui U.T., Finlayson K., Edwards H. (2018). Risk factors for infection in patients with chronic leg ulcers: A survival analysis. Int. J. Clin. Pract..

[18] Michael J.E., Maier M. (2016). Lower extremity ulcers. Vasc. Med..

[19] Salenius J.E., Suntila M., Ahti T., Huhtala H., Vaalasti A., Salmi T.T., Kimpimäki T. (2021). Long-term mortality among patients with chronic ulcers. Acta Derm. Venereol..

[20] Olin J.W., Beusterien K.M., Childs M.B., Seavey C., McHugh L., Griffiths R.I. (1999). Medical costs of treating venous stasis ulcers: Evidence from a retrospective cohort study. Vasc. Med..

[21] Curti N., Merli Y., Zengarini C., Starace M., Rapparini L., Marcelli E., Carlini G., Buschi D., Castellani G.C., Piraccini B.M. (2024). Automated prediction of photographic wound assessment tool in chronic wound images. J. Med. Syst..

[22] Barakat-Johnson M., Jones A., Burger M., Leong T., Frotjold A., Randall S., Kim B., Fethney J., Coyer F. (2022). Reshaping wound care: Evaluation of an artificial intelligence app to improve wound assessment and management amid the COVID-19 pandemic. Int. Wound J..

[23] Kolasa K., Admassu B., Hołownia-Voloskova M., Kędzior K.J., Poirrier J.E., Perni S. (2024). Systematic reviews of machine learning in healthcare: A literature review. Expert Rev. Pharmacoecon. Outcomes Res..

[24] Frasca M., La Torre D., Pravettoni G., Cutica I. (2024). Explainable and interpretable artificial intelligence in medicine: A systematic bibliometric review. Discov. Artif. Intell..

[25] Thomsen K., Iversen L., Titlestad T.L., Winther O. (2020). Systematic review of machine learning for diagnosis and prognosis in dermatology. J. Dermatolog. Treat..

[26] Schaarup C., Pape-Haugaard L.B., Hejlesen O.K. (2018). Models used in clinical decision support systems supporting healthcare professionals treating chronic wounds: Systematic literature review. JMIR Diabetes.

[27] Chen M.Y., Cao M.Q., Xu T.Y. (2024). Progress in the application of artificial intelligence in skin wound assessment and prediction of healing time. Am. J. Transl. Res..

[28] Reifs Jiménez D., Casanova-Lozano L., Grau-Carrión S., Reig-Bolaño R. (2025). Artificial intelligence methods for diagnostic and decision-making assistance in chronic wounds: A systematic review. J. Med. Syst..

[29] Pugalenthi L.S., Garapati C., Maddukuri S., Kanwal F., Kumar J., Asadimanesh N., Dadwal S., Ahluwalia V., Senapati S.G., Arunachalam S.P. (2025). From data to decisions: AI in varicose veins—Predicting, diagnosing, and guiding effective management. J. Vasc. Dis..

[30] Ahmed A., Xi R., Hou M., Shah S.A., Hameed S. (2023). Harnessing big data analytics for healthcare: A comprehensive review of frameworks, implications, applications, and impacts. IEEE Access.

[31] Anisuzzaman D.M., Patel Y., Rostami B., Niezgoda J., Gopalakrishnan S., Yu Z. (2022). Multi-modal wound classification using wound image and location by deep neural network. Sci. Rep..

[32] Cukjati D., Robnik-Šikonja M., Reberšek S., Kononenko I., Miklavčič D. (2001). Prognostic factors in the prediction of chronic wound healing by electrical stimulation. Med. Biol. Eng. Comput..

[33] Robnik-Šikonja M., Cukjati D., Kononenko I. (2003). Comprehensible evaluation of prognostic factors and prediction of wound healing. Artif. Intell. Med..

[34] Jung K., Covington S., Sen C.K., Januszyk M., Kirsner R.S., Gurtner G.C., Shah N.H. (2016). Rapid identification of slow healing wounds. Wound Repair Regen..

[35] Fife C.E., Horn S.D. (2020). The wound healing index for predicting venous leg ulcer outcome. Adv. Wound Care.

[36] Kurd S.K., Hoffstad O.J., Bilker W.B., Margolis D.J. (2009). Evaluation of the use of prognostic information for the care of individuals with venous leg ulcers or diabetic neuropathic foot ulcers. Wound Repair Regen..

[37] Wu J., Chen X.Y., Zhang H., Xiong L.D., Lei H., Deng S.H. (2019). Hyperparameter optimization for machine learning models based on Bayesian optimization. J. Electron. Sci. Technol..

[38] Chino D.Y.T., Scabora L.C., Cazzolato M.T., Jorge A.E.S., Traina C., Traina A.J.M. ICARUS: Retrieving skin ulcer images through bag-of-signatures. Proceedings of the 2018 IEEE 31st International Symposium on Computer-Based Medical Systems.

[39] Blanco G., Traina A.J.M., Traina C., Azevedo-Marques P.M., Jorge A.E.S., de Oliveira D., Bedo M.V.N. (2020). A superpixel-driven deep learning approach for the analysis of dermatological wounds. Comput. Methods Programs Biomed..

[40] Gupta R., Goldstone L., Eisen S., Ramachandram D., Cassata A., Fraser R.D.J., Ramirez-GarciaLuna J.L., Bartlett R., Allport J. (2024). Towards an AI-based objective prognostic model for quantifying wound healing. IEEE J. Biomed. Health Inform..

[41] Ramachandram D., Ramirez-GarciaLuna J.L., Fraser R.D.J., Martínez-Jiménez M.A., Arriaga-Caballero J.E., Allport J. (2022). Fully automated wound tissue segmentation using deep learning on mobile devices: Cohort study. JMIR mHealth uHealth.

[42] Lu H., Li B., Zhu J., Li Y., Li Y., Xu X., He L., Li X., Li J., Serikawa S. (2017). Wound intensity correction and segmentation with convolutional neural networks. Concurr. Computat. Pract. Exper..

[43] Galushka M., Zheng H., Patterson D., Bradley L. Case-based tissue classification for monitoring leg ulcer healing. Proceedings of the 18th IEEE Symposium on Computer-Based Medical Systems (CBMS’05).

[44] Patel Y., Shah T., Dhar M.K., Zhang T., Niezgoda J., Gopalakrishnan S., Yu Z. (2024). Integrated image and location analysis for wound classification: A deep learning approach. Sci. Rep..

[45] Rostami B., Anisuzzaman D.M., Wang C., Gopalakrishnan S., Niezgoda J., Yu Z. (2021). Multiclass wound image classification using an ensemble deep CNN-based classifier. Comput. Biol. Med..

[46] Godeiro V., Neto J.S., Carvalho B., Santana B., Ferraz J., Gama R. Chronic wound tissue classification using convolutional networks and color space reduction. Proceedings of the 2018 IEEE International Workshop on Machine Learning for Signal Processing.

[47] Lo Z.J., Mak M.H.W., Liang S., Chan Y.M., Goh C.C., Lai T., Tan A., Thng P., Rodriguez J., Weyde T. (2024). Development of an explainable artificial intelligence model for Asian vascular wound images. Int. Wound J..

[48] Howell R.S., Liu H.H., Khan A.A., Woods J.S., Lin L.J., Saxena M., Saxena H., Castellano M., Petrone P., Slone E. (2021). Development of a method for clinical evaluation of artificial intelligence-based digital wound assessment tools. JAMA Netw. Open.

[49] Chino D.Y.T., Scabora L.C., Cazzolato M.T., Jorge A.E.S., Traina C., Traina A.J.M. (2020). Segmenting skin ulcers and measuring the wound area using deep convolutional networks. Comput. Methods Programs Biomed..

[50] Ohura N., Mitsuno R., Sakisaka M., Terabe Y., Morishige Y., Uchiyama A., Okoshi T., Shinji I., Takushima A. (2019). Convolutional neural networks for wound detection: The role of artificial intelligence in wound care. J. Wound Care.

[51] Anisuzzaman D.M., Patel Y., Niezgoda J.A., Gopalakrishnan S., Yu Z. (2022). A mobile app for wound localization using deep learning. IEEE Access.

[52] Kolesnik M., Fexa A. Segmentation of wounds in the combined color-texture feature space. Proceedings of the SPIE 5370, Medical Imaging 2004: Image Processing.

[53] Kolesnik M., Fexa A. How robust is the SVM wound segmentation?. Proceedings of the 7th Nordic Signal Processing Symposium—NORSIG 2006.

[54] Curti N., Merli Y., Zengarini C., Giampieri E., Merlotti A., Dall’Olio D., Marcelli E., Bianchi T., Castellani G. (2022). Effectiveness of semi-supervised active learning in automated wound image segmentation. Int. J. Mol. Sci..

[55] Yadav M.K., Manohar D.D., Mukherjee G., Chakraborty C. (2013). Segmentation of chronic wound areas by clustering techniques using selected color space. J. Med. Imaging Health Inform..

[56] Silva W.S., Jasbick D.L., Wilson R.E., Azevedo-Marques P.M., Traina A.J.M., Santos L.F.D., Jorge A.E.S., de Oliveira D., Bedo M.V.N. A two-phase learning approach for the segmentation of dermatological wounds. Proceedings of the 2019 IEEE 32nd International Symposium on Computer-Based Medical Systems (CBMS).

[57] Maity M., Dhane D., Bar C., Chakraborty C., Chatterjee J., Bhattacharyya S., Chaki N., Konar D., Chakraborty U., Singh C. (2018). Assessment of segmentation techniques for chronic wound surface area detection. Advanced Computational and Communication Paradigms, Advances in Intelligent Systems and Computing.

[58] Dhane D.M., Krishna V., Achar A., Bar C., Sanyal K., Chakraborty C. (2016). Spectral clustering for unsupervised segmentation of lower extremity wound beds using optical images. J. Med. Syst..

[59] Seixas J.L., Barbon S., Mantovani R.G. Pattern recognition of lower member skin ulcers in medical images with Machine Learning Algorithms. Proceedings of the 2015 IEEE 28th International Symposium on Computer-Based Medical Systems.

[60] Liu Z., Agu E., Pedersen P., Lindsay C., Tulu B., Strong D. (2023). Chronic wound image augmentation and assessment using semi-supervised progressive multi-granularity EfficientNet. IEEE Open J. Eng. Med. Biol..

[61] Liu Z., Agu E., Pedersen P., Lindsay C., Tulu B., Strong D. (2021). Comprehensive assessment of fine-grained wound images using a patch-based CNN with context-preserving attention. IEEE Open J. Eng. Med. Biol..

[62] Monroy B., Bacca J., Sanchez K., Arguello H., Castillo S. Two-step deep learning framework for chronic wounds detection and segmentation: A case study in Colombia. Proceedings of the 2021 XXIII Symposium on Image, Signal Processing and Artificial Vision, STSIVA 2021.

[63] Shi Q., Chen W., Pan Y., Yin S., Fu Y., Mei J., Xue Z. (2018). An automatic classification method on chronic venous insufficiency images. Sci. Rep..

[64] Barulina M., Sanbaev A., Okunkov S., Ulitin I., Okoneshnikov I. (2022). Deep learning approaches to automatic chronic venous disease classification. Mathematics.

[65] Stefanelli A., Zahia S., Chanel G., Niri R., Pichon S., Probst S. (2025). Developing an AI-powered wound assessment tool: A methodological approach to data collection and model optimization. BMC Med. Inform. Decis. Mak..

[66] Niri R., Zahia S., Stefanelli A., Sharma K., Probst S., Pichon S., Chanel G. (2025). Wound segmentation with U-Net using a dual attention mechanism and transfer learning. J. Imaging Inform. Med..

[67] Mukherjee R., Manohar D.D., Das D.K., Achar A., Mitra A., Chakraborty C. (2014). Automated tissue classification framework for reproducible chronic wound assessment. BioMed Res. Int..

[68] Antunović A., Nyarko E.K., Filko D. Wound tissue classification: A comparative analysis of deep neural network models. Proceedings of the 2024 International Conference on Smart Systems and Technologies (SST).

[69] Rajathi V., Bhavani R.R., Wiselin Jiji G. (2019). Varicose ulcer(C6) wound image tissue classification using multidimensional convolutional neural networks. Imaging Sci. J..

[70] Wannous H., Lucas Y., Treuillet S. (2011). Enhanced assessment of the wound-healing process by accurate multiview tissue classification. IEEE Trans. Med. Imaging.

[71] Jaslin A.M. (2018). Tissue classification of varicose ulcer based on features using an efficient CSVM classifier. Int. J. Emerg. Technol. Comput. Sci. Electron..

[72] Chakraborty C. (2019). Computational approach for chronic wound tissue characterization. Inform. Med. Unlocked.

[73] Nejati H., Ghazijahani H.A., Abdollahzadeh M., Malekzadeh T., Cheung N.M., Lee K.H., Low L.L. Fine-grained wound tissue analysis using deep neural network. Proceedings of the 2018 IEEE International Conference on Acoustics, Speech and Signal Processing (ICASSP).

[74] Pereira S.M., Frade M.A.C., Rangayyan R.M., de Azevedo Marques P.M. Classification of dermatological ulcers based on tissue composition and color texture features. Proceedings of the 4th International Symposium on Applied Sciences in Biomedical and Communication Technologies (ISABEL ’11).

[75] Bedo M.V.N., Santos L.F.D., Oliveira W.D., Blanco G., Traina A.J.M., Frade M.A.C., Azevedo-Marques P.M., Traina C. Color and texture influence on computer-aided diagnosis of dermatological ulcers. Proceedings of the 2015 IEEE 28th International Symposium on Computer-Based Medical Systems.

[76] Zheng H., Bradley L., Patterson D., Galushka M., Winder J. New protocol for leg ulcer tissue classification from colour images. Proceedings of the 26th Annual International Conference of the IEEE Engineering in Medicine and Biology Society.

[77] Wannous H., Treuillet S., Lucas Y. Supervised tissue classification from color images for a complete wound assessment tool. Proceedings of the 2007 29th Annual International Conference of the IEEE Engineering in Medicine and Biology Society.

[78] Wannous H., Treuillet S., Lucas Y. (2010). Robust tissue classification for reproducible wound assessment in telemedicine environments. J. Electron. Imaging.

[79] Sarp S., Kuzlu M., Pipattanasomporn M., Guler O. (2021). Simultaneous wound border segmentation and tissue classification using a conditional generative adversarial network. J. Eng..

[80] Kabir M.A., Roy N., Hossain M.E., Featherston J., Ahmed S. (2025). Deep learning for wound tissue segmentation: A comprehensive evaluation using a novel dataset. arXiv.

[81] Aguirre Nilsson C., Velic M. (2018). Classification of Ulcer Images Using Convolutional Neural Networks. Master’s Thesis.

[82] Huang P.H., Pan Y.H., Luo Y.S., Chen Y.F., Lo Y.C., Chen T.P., Perng C.K. (2023). Development of a deep learning-based tool to assist wound classification. J. Plast. Reconstr. Aesthet. Surg..

[83] Jahangir M.Z.B., Akter S., Nasim M.A.A., Gupta K.D., George R. (2024). Deep learning for automated wound classification and segmentation. arXiv.

[84] Malihi L., Hüsers J., Richter M.L., Moelleken M., Przysucha M., Busch D., Heggemann J., Hafer G., Wiemeyer S., Heidemann G. (2022). Automatic wound type classification with convolutional neural networks. Stud. Health Technol. Inform..

[85] Neuwieser H., Jami N.V.S.J., Meier R.J., Liebsch G., Felthaus O., Klein S., Schreml S., Berneburg M., Prantl L., Leutheuser H. (2025). Interpreting venous and arterial ulcer images through the Grad-CAM lens: Insights and implications in CNN-based wound image classification. Diagnostics.

[86] Mousa R., Matbooe E., Khojasteh H., Bengari A., Vahediahmar M. (2025). Multi-modal wound classification using wound image and location by Xception and Gaussian Mixture Recurrent Neural Network (GMRNN). arXiv.

[87] Ullah S., Javed A., Aljasem M., Saudagar A.K.J. (2025). Eff-ReLU-Net: A deep learning framework for multiclass wound classification. BMC Med. Imaging.

[88] Mombini H., Tulu B., Strong D., Agu E., Nguyen H., Lindsay C., Loretz L., Pedersen P., Dunn R., Hofmann S., Müller O., Rossi M. (2020). Design of a machine learning system for prediction of chronic wound management decisions. Designing for Digital Transformation, Co-Creating Services with Citizens and Industry, DESRIST 2020, Lecture Notes in Computer Science.

[89] Nguyen H., Agu E., Tulu B., Strong D., Mombini H., Pedersen P., Lindsay C., Dunn R., Loretz L. (2020). Machine learning models for synthesizing actionable care decisions on lower extremity wounds. Smart Health.

[90] Estrela V.V., Herrmann A.E., Cruz-Cunha M.M., Miranda I.M., Martinho R., Rijo R. (2016). Content-Based Image Retrieval (CBIR) in Remote Clinical Diagnosis and Healthcare. Encyclopedia of E-Health and Telemedicine.

[91] Müller H., Michoux N., Bandon D., Geissbuhler A. (2004). A review of content-based image retrieval systems in medical applications-clinical benefits and future directions. Int. J. Med. Inform..

[92] Dorileo E.A., Frade M.A., Roselino A.M., Rangayyan R.M., Azevedo-Marques P.M. Color image processing and content-based image retrieval techniques for the analysis of dermatological lesions. Proceedings of the 2008 30th Annual International Conference of the IEEE Engineering in Medicine and Biology Society (EMBS 2008).

[93] Pereyra L.C., Pereira S.M., Souza J.P., Frade M.A.C., Rangayyan R.M., Azevedo-Marques P.M. (2014). Characterization and pattern recognition of color images of dermatological ulcers: A pilot study. Comput. Sci. J. Mold..

[94] Blanco G., Bedo M.V.N., Cazzolato M.T., Santos L.F.D., Jorge A.E.S., Traina C., Azevedo-Marques P.M., Traina A.J.M. A label-scaled similarity measure for content-based image retrieval. Proceedings of the 2016 IEEE International Symposium on Multimedia (ISM).

[95] Jaganathan Y., Sanober S., Aldossary S.M.A., Aldosari H. (2023). Validating wound severity assessment via region-anchored convolutional neural network model for mobile image-based size and tissue classification. Diagnostics.

[96] Wang C., Yan X., Smith M., Kochhar K., Rubin M., Warren S.M., Wrobel J., Lee H. A unified framework for automatic wound segmentation and analysis with deep convolutional neural networks. Proceedings of the 2015 37th Annual International Conference of the IEEE Engineering in Medicine and Biology Society (EMBC 2015).

[97] Chakraborty C., Gupta B., Ghosh S.K., Das D.K., Chakraborty C. (2016). Telemedicine supported chronic wound tissue prediction using classification approaches. J. Med. Syst..

[98] Li F., Wang C., Liu X., Peng Y., Jin S. (2018). A composite model of wound segmentation based on traditional methods and deep neural networks. Comput. Intell. Neurosci..

[99] Quemy A. (2020). Two-stage optimization for machine learning workflow. Inf. Syst..

[100] Subramani B., Veluchamy M. (2023). Bilateral tone mapping scheme for color correction and contrast adjustment in nearly invisible medical images. Color Res. Appl..

[101] Subramani B., Veluchamy M., Bhandari A.K. (2023). Optimal fuzzy intensification system for contrast distorted medical images. IEEE Trans. Emerg. Top. Comp. Intell..

[102] Yang H., Ma Y., Khan F.G., Khan A., Ali F., AlZubi A.A., Zeng H. (2024). Survey: Application and analysis of generative adversarial networks in medical images. Artif. Intell. Rev..

[103] Zhao C., Guo Y., Li L., Yang M. (2024). Non-invasive techniques for wound assessment: A comprehensive review. Int. Wound J..

[104] Palani P., Subramani B., Veluchamy M. (2026). Adaptive degradation-aware medical image enhancement for multi-modal diagnostics. Biomed. Signal Process. Control.

[105] Subramani B., Veluchamy M. (2025). Contrast-Aware multiscale decomposition and noise suppression framework for reliable clinical measurements in degraded medical images. Measurement.

[106] Cap Q.H., Fukuda A., Iyatomi H. (2025). A practical framework for unsupervised structure preservation medical image enhancement. Biomed. Signal Process. Control.

[107] Griffa D., Natale A., Merli Y., Starace M., Curti N., Mussi M., Castellani G., Melandri D., Piraccini B.M., Zengarini C. (2024). Artificial intelligence in wound care: A narrative review of the currently available mobile apps for automatic ulcer segmentation. BioMedInformatics.

[108] Sirocchi C., Bogliolo A., Montagna S. (2024). Medical-informed machine learning: Integrating prior knowledge into medical decision systems. BMC Med Inform. Decis. Mak..

